# Methods for grafting *Arabidopsis thaliana* and *Eutrema salsugineum*

**DOI:** 10.1186/s13007-019-0477-x

**Published:** 2019-08-13

**Authors:** Yan Li, Wei Sun, Fulin Liu, Jin Cheng, Xiaojie Zhang, Hui Zhang, Yanxiu Zhao

**Affiliations:** Shandong Provincial Key Laboratory of Plant Stress Research, College of Life Science, Shandong Normal University, Jinan, 250014 Shandong China

**Keywords:** *Arabidopsis thaliana*, Cut-in grafting, *Eutrema salsugineum* (*Thellungiella salsuginea*), Sterile, ICP-OES

## Abstract

**Background:**

Grafting, an ancient agronomic technique, is an artificial mode of asexual reproduction in plants. Recently, grafting research has gradually shifted from modifying agronomic traits to the study of molecular mechanism. Grafting is an excellent tool to study long-range signaling processes in plants. And the grafting between species will help elucidate the molecular mechanisms underlying contrasting differences between different species. *Arabidopsis thaliana* is a salt-sensitive model glycophyte and *Eutrema salsugineum* (previously *Thellungiella salsuginea*, salt cress) is a salt-tolerant model halophyte. Successful grafting of these two model plants will help further study the physiological and molecular mechanisms underlying salt tolerance in plants. The aim of this study was to demonstrate two sterile micro-grafting methods for *Arabidopsis* and salt cress.

**Results:**

We developed the methods for sterile grafting between *A. thaliana* and *E. salsugineum*; this is the first report on inter-generic grafting between *Arabidopsis* and *Eutrema*. The method involves cut-in grafting under sterile conditions. The grafted plant part was placed in half strength Murashige and Skoog medium with 1% agar and 1% sugar, and then cultured vertically with 22 °C/18 °C short-day/night cycles. The plants were then transferred to half strength Hoagland nutrient solution for hydroponics. The reported method is simple and easy to operate. Self-grafted *Arabidopsis*–*Arabidopsis* and *Eutrema*–*Eutrema* plants were used as controls, which were obtained with an improved hypocotyl-cutting grafting method. Ion contents in grafted plants were detected by inductively coupled plasma optical emission spectroscopy. The results showed that the ion content in salt cress and *Arabidopsis* changed to different degrees after grafting.

**Conclusions:**

The inter-species grafting technique described here makes it possible to study hybrid plants between *Arabidopsis* and *Eutrema* and will contribute to further understanding of long-distance communications in plants. This technique also provides a reference for improving plant varieties using grafting, such as gardening plants, as well as fruit and vegetable crops.

## Background

Grafting, an old agronomic technique, is a type of vegetative/asexual propagation of plants. It is usually carried out by exploiting callus growth after plant injury. Grafting is still a common technique in the field of gardening. Grafting has been used in agriculture since > 2 000 years BCE (Before the Common Era) [1, 2] in China. It is widely used in agriculture, and the cultivation of forest trees, and horticultural plants. For instance, cultivars with pathogen resistance or stress tolerance are usually used as rootstocks to improve the growth vigor of above-ground cultivars with high economic values. Grafting has also been used to break the juvenile stage in order to promote flowering in woody species. Compared to genetically modified crops, grafted plants are more palatable to the public [3]. Grafting is also a useful technique in plant scientific research. Considerable efforts in this direction started in the 70 s and 80 s of the last century. However, with the rapid development of biotechnology, the focus of grafting research has gradually shifted from improving agronomic traits to the study of molecular mechanism. Studies have demonstrated that grafting is an excellent tool in elucidating mechanisms involved in long-range signaling processes within plants, such as those involved in flowering [4–7], auxin regulation [8], heavy metal tolerance [9], small RNA movement [10, 11], nutrition state [10], and nuclear silencing [12, 13].

It is well known that the factors affecting graft survival rates mainly depend on the compatibility of the rootstock and scion. Compatibility refers to the similarity between the rootstock and scion with respect to structural, genetic, and physiological characteristics, and the ability to adapt to each other after grafting. The compatibility of grafts from the same plants is the strongest; the farther the relationship between plants, the weaker the compatibility between them. It is difficult to graft plants from different genera. Besides the compatibility of scions and rootstocks, the vigor of rootstock and scion is also an important factor affecting graft survival. The survival rate of grafted plants also varies with species, and the survival rate of the grafted body is different even in the same species at different ages. Furthermore, environmental factors such as temperature, humidity, and light intensity can also affect the survival rate of grafted plants. In conclusion, there are many factors that influence graft survival rate.

*Arabidopsis thaliana* and *Eutrema salsugineum* (*Thellungiella salsuginea*) belong to the same family Cruciferae but different genera. *Arabidopsis thaliana* is used as a model plant in genetic studies of dicotyledonous plants. *Eutrema Salsugineum* (aka. Salt cress) is an excellent salt-tolerant plant, and the genome of *E. salsugineum* has been completed [14]. Approximately 95% of the genes in *E. salsugineum* are similar to those expressed in *Arabidopsis* [15], and most of the amino acid sequences are the same as those of *Arabidopsis* [14]. *Eutrema salsugineum* has a relatively small nuclear genome, approximately two-times of that of *A. thaliana* [16]. All the ecotypes of *E. salsugineum* show resistance to a range of environmental stresses such as cold, drought and oxidative stresses [17]. *E. salsugineum* has gained attention owing to its extreme salt-tolerance. Therefore, salt cress was suggested as a model halophyte for studying salt tolerance more than 20 years ago [15, 18–20]. This has opened a new window for research covering a wide range of aspects, including not only abiotic stress [21], but also photosynthesis [22], stress-related protein function [23], and surface wax production [24] using *E. salsugineum* as a model plant. In *Arabidopsis*, several grafting approaches have been developed to investigate systemic signaling [9, 25–31]. However, to the best of our knowledge, there is no report on grafting methods for *E. salsugineum*, or on methods for grafting between *Arabidopsis* and *Eutrema*. The difficulty of grafting between these plants is significantly high. Successful grafting of these two model plants will help provide crucial insights into molecular mechanisms underlying plant stress tolerance. Here we demonstrate two sterile micro-grafting methods for inter-generic grafting between *Arabidopsis* and *Eutrema*, with the advantages of simplicity and high survival rate.

### Grafting methods

**Fig. 1:**
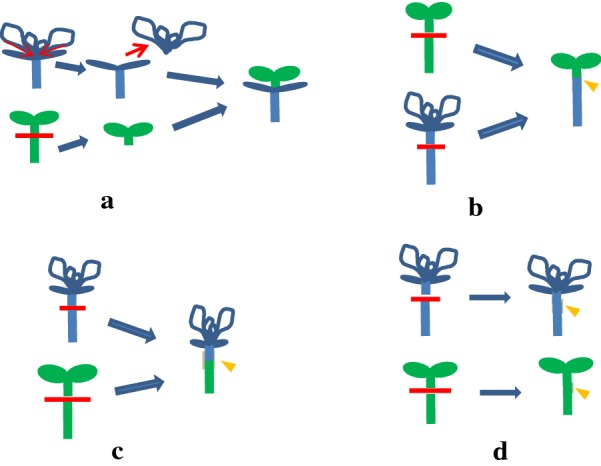
Diagram of grafting technique. **a**, **b** Two Different Grafting Methods of *Arabidopsis thaliana* Grafted on *Eutrema. Salsugineum* (A/E), cut-in grafting (**a**) and hypocotyl–hypocotyl flat-surface cutting (**b**). **c** E/A (*E. salsugineum* as scions were grafted onto *A. thaliana* as stocks. (**d**) Self-grafting of *E. salsugineum* (E/E) and *A. thaliana* (A/A). The red line indicates the cutting position. Orange arrowheads point to grafting junction between the shoot and root tissue with silicone tubing support (light grey). Red arrows in (**a**) denote cutting or removal parts. Differences in color show different plants, blue for *E. salsugineum* and green for *A. thaliana*. Roots are not shown here

In the present study, we focus on two efficient sterile seedling micro-grafting protocols between *Arabidopsis*, a salt-sensitive plant, and salt cress, a salt-tolerant plant. One method was hypocotyl–hypocotyl flat-surface cutting of the seedlings, as described by Seung [26] and Turnbull et al. [25] with some modifications. The other method was cut-in grafting, which we will demonstrate herein. The abbreviation A/E used herein indicates that *A. thaliana* scions were grafted onto *E. salsugineum* stocks, and conversely E/A indicates that *E. salsugineum* scions were grafted onto *A. thaliana* stocks. In a control experiment, self-grafting of *A. thaliana* and *E. salsugineum* was performed. The self-grafted seedlings of *A. thaliana* are referred to as A/A and the self-grafted seedlings of *E. salsugineum* are referred to as E/E. Optimized grafting methods were applied to different rootstocks and scions. The method of cut-in grafting was used for A/E (Fig. 1a) and the other method, i.e., hypocotyl–hypocotyl flat-surface cutting was used for A/A, E/E, and E/A (Fig. 1c, d). It is relatively easy to achieve successful graft using the conventional grafting method (i.e., hypocotyl–hypocotyl flat-surface cutting), and the subsequent growth of grafted plants is usually satisfactory. Therefore, we will not discuss this method herein.

### Materials and growth conditions

*Arabidopsis thaliana* Columbia-0 (Col) seeds and *E. salsugineum* (Shandong, China) seeds used in this study are preserved and propagated in our laboratory. The plants were grown under long-day (LD 16/8 h light/dark) or short-day (SD 8/16 h light/dark) light conditions, with 22 °C/18 °C day/night cycles and 100 μmol m^−2^s^−1^ light intensity.

### Grafting procedure

The step-by-step protocol of cut-in grafting method applied for A/E was as follows:

Preparation of rootstock and scion materials: salt cress seeds was disinfected with 0.5% NaClO (sodium hydrochlorite) for 6 min followed by 4–5 rinses in distilled water, and subsequently sowed under sterile conditions on half strength Murashige and Skoog (MS) medium containing 1.0% agar and 1% sugar. One week after low temperature (4 °C) stratification treatment, the seedlings were transferred to SD conditions and vertically cultured with light for 10–15 days (or 7–10 days after germination) as rootstock seedlings. For the germination of *Arabidopsis* seeds, the sterilization method and growth conditions were the same as those of *E. salsugineum*, except the low temperature (4 °C) stratification treatment, which was for 2–3 days. The optimum age for grafting *Arabidopsis* seedlings is 3–5 days. Because the germination time and growth rate of the two species are different, the planting times were adjusted accordingly. Usually *Arabidopsis* should be planted after the germination of salt cress seeds.

**Fig. 2:**
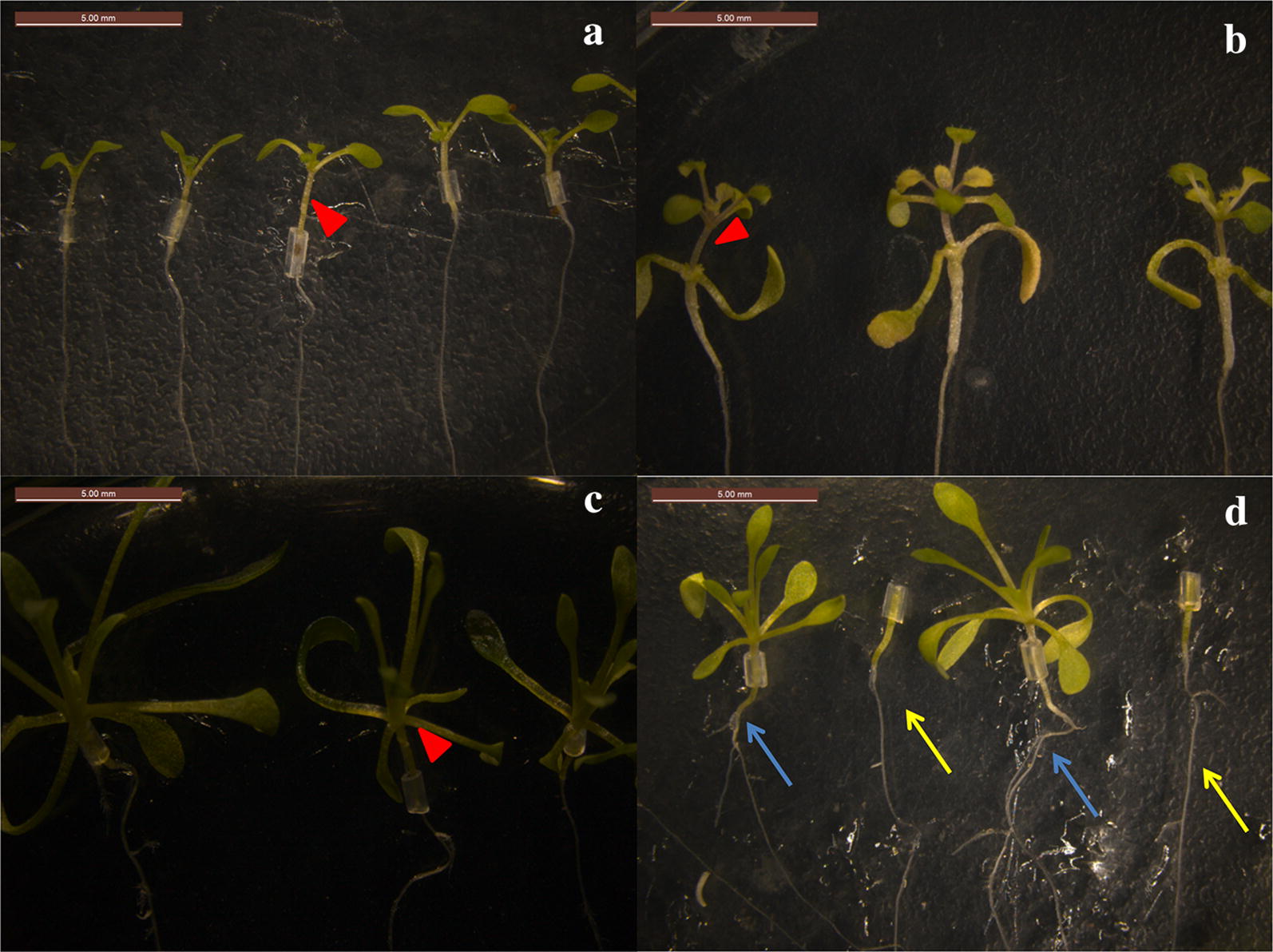
Succeed grafted seedlings. **a** Self-grafting of *A. thaliana* (A/A). **b** A/E grafted seedlings. **c** Self-grafting of *E. salsugineum* (E/E). **d** E/A grafted seedlings. Red arrows in **a**–**c** denote grafting sites. *Arabidopsis thaliana* roots as rootstocks are shown by arrows of different colors in **d**, blue arrows indicate the roots after grafting, and yellow arrows indicate the roots before grafting

1. Grafting under sterile conditions (Fig. 1a): The grafting procedure was performed under anatomical microscopes in a sterile hood. *E. salsugineum* seedlings of uniform size were moved using tweezers to new 1.0% half strength MS agar medium. To be used as rootstocks, the true leaves and meristems were removed from salt cress seedlings with a stainless steel double-edged blade razor, while the cotyledons were retained. Under the same sterile conditions, the hypocotyl of *A. thaliana* seedings was cut transversely at approximately one-fourth of the height from the top (typically 2–3 mm) and the upper part of the seedling was used as a scion. Cutting of *A. thaliana* as scions can be carried out directly in the original medium plates or in the plates where the salt cress has been prepared as rootstocks. The *A. thaliana* scion was grafted onto salt cress without any fixtures because the tiny amount of liquid at the cutting surfaces of the two parts is sufficient to hold them together. Afterwards, the Petri dish was swiftly but carefully sealed with parafilm to preserve humidity. Please take caution to make sure the leaves are naturally extended to prevent possible dislodging due to plant growth.
2. Culturing of grafted body: The grafted A/E complex was placed under SD conditions in a Percival growth cabinet for 6–8 days.
3. Transplantation of grafted seedlings (Fig. 2): Six to eight days after the grafting, the interface of butt joint was examined under an anatomic microscope. The successfully graft should have solid connection between the scion and the stock by this time. The seedlings were then transferred to a hydroponic system or nutritious soil per requirement. After the transplanting, attention should be paid to cut off adventitious roots in time, if the adventitious roots occur.

### Seed surface disinfection

In the cut-in grafting method, the surface of seeds was disinfected directly using 0.5% sodium hydrochloride (NaClO) for 6–8 min, and then washed with sterile water for 4–6 times. This method avoids the use of alcohol for sterilization. Moreover, the sterilization method with NaClO is simple, thereby reducing secondary pollution, and the seeds can germinate normally. It also avoids non-germination of seeds because of over sterilization, which affects seed germination and subsequent analyses. As long as the concentration of NaClO is controlled properly, over-sterilization will not occur even if the sterilization lasts for several minutes.

### Seedling age

In the cut-in grafting method, slightly older *E. salsugineum* seedlings (7–10 days after sprouting) with lateral roots, can better meet the fast growth characteristics of their scions (*A. thaliana*), this also reduces the formation of adventitious roots, greatly improves the survival rate of grafts and ensure the vigorous growth of grafted seedlings at the later stage (Table 1). We found that 3–5-day-old seedlings of *Arabidopsis* were favorable to improve the survival rate of grafted seedlings; it also supports the vigorous growth of grafted seedlings (Table 1). This may be because the life cycle of *A. thaliana* is short, compared to that of *E. salsugineum.* Therefore the use of older *A. thaliana* seedlings as scions resulted in low survival rates; even if the A/E grafts survived, the growth at later stages was poor and early flowering is usually observed.

**Table 1:** Varying the age of the seedlings on A/E grafting

Scion age	Rootstock age	Total grafts	Grafting success	Success rate (%)	Later growth
3–5	3–5	100	50	50	Grow weak
	7–10	100	96	96	Grow strong
	11–15	100	80	80	Grow strong
6–8	3–5	100	20	20	Grow weak
	7–10	100	70	70	General growth
	11–15	100	60	60	General growth

### Flat-surface cutting method

Multiple types of grafting methods have been reported for *A. thaliana* [26, 27, 30, 31]. In the present study, the method of cut-in grafting was only used for A/E grafts and the method of hypocotyl–hypocotyl flat-surface cutting was used for A/A, E/E, and E/A (Fig. 1c, d). The two methods require the same culturing conditions except for the cutting site. Seedlings aged 3–5 days were used for A/A self-grafting. For E/A grafting, 7 to 10-day-old seedlings of *A. thaliana* as rootstocks are more beneficial for the survival of grafts. Similarly, 7–10 day old Seedlings were used for E/E self-grafting. We found that both the survival rate and later growth of A/E seedlings were significantly different between the two grafting methods (Fig. 1a, b and Table 2). The cut-in method is more satisfactory for A/E grafting. The main reason might be the fast growth rate of *A. thaliana* is better accommodated using this method. When traditional flat-surface cutting was used for A/E grafting, adventitious roots from the scion appear before the callus formation at the junction, affecting the success rate of grafted plants, because the growth of salt cress is slower than that of *A. thaliana*. In comparison the success rate of the cut-in method is almost 100% as long as the cut is smooth and two parts tightly placed.

**Table 2:** Effects of two grafting methods on A/E grafting

Grafting method	Total grafts	Grafting success	Success rate (%)	Later growth
Hypocotyl–hypocotyl flat-surface cutting	80	10	12.5	Grow weak
Cut-in grafting	80	68	85	Grow strong

## Results

### Successfully grafted seedlings

After successful grafting of scions and rootstocks, it was apparent that the above-ground and under-ground parts promote the growth of each other, whereas the un-grafted rootstocks and scions remain basically unchanged (Fig. 2d).

### Characterization of growth

**Fig. 3:**
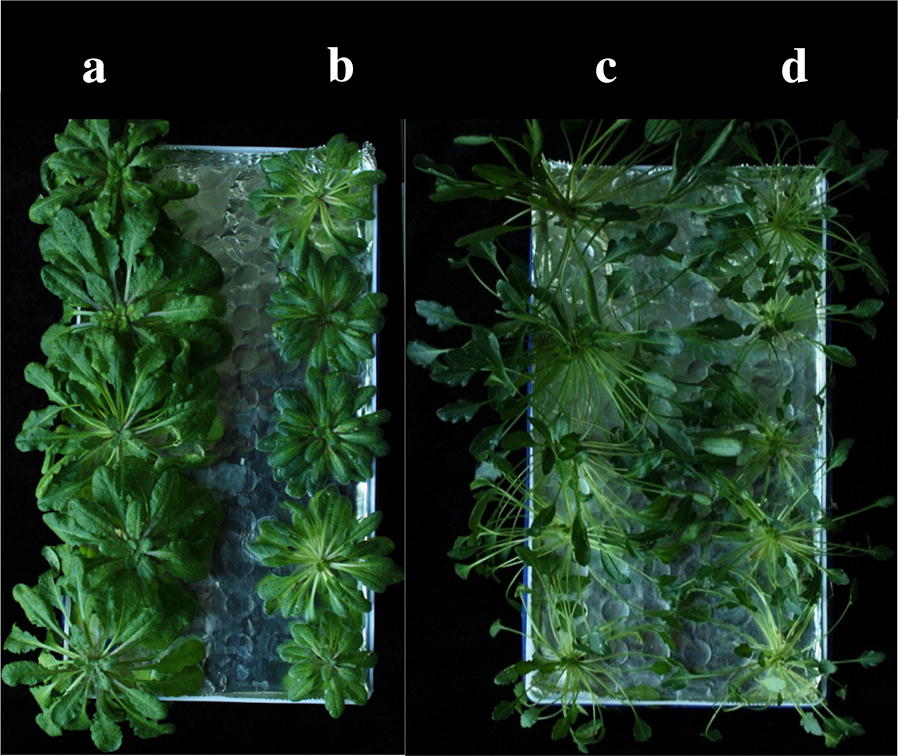
Two-month grafted seedlings (**a**, **b**) different rootstocks with the same scion (*Arabidopsis*), as do (**c**, **d**) except that the scion is Salt Cress. **a** Rootstock is *Arabidopsis thaliana*(A/A), **b** Rootstock is *E. salsugineum* (A/E), **c** Rootstock is *Arabidopsis thaliana* (E/A), **d** Rootstock is *E. salsugineum* (E/E)

**Fig. 4:**
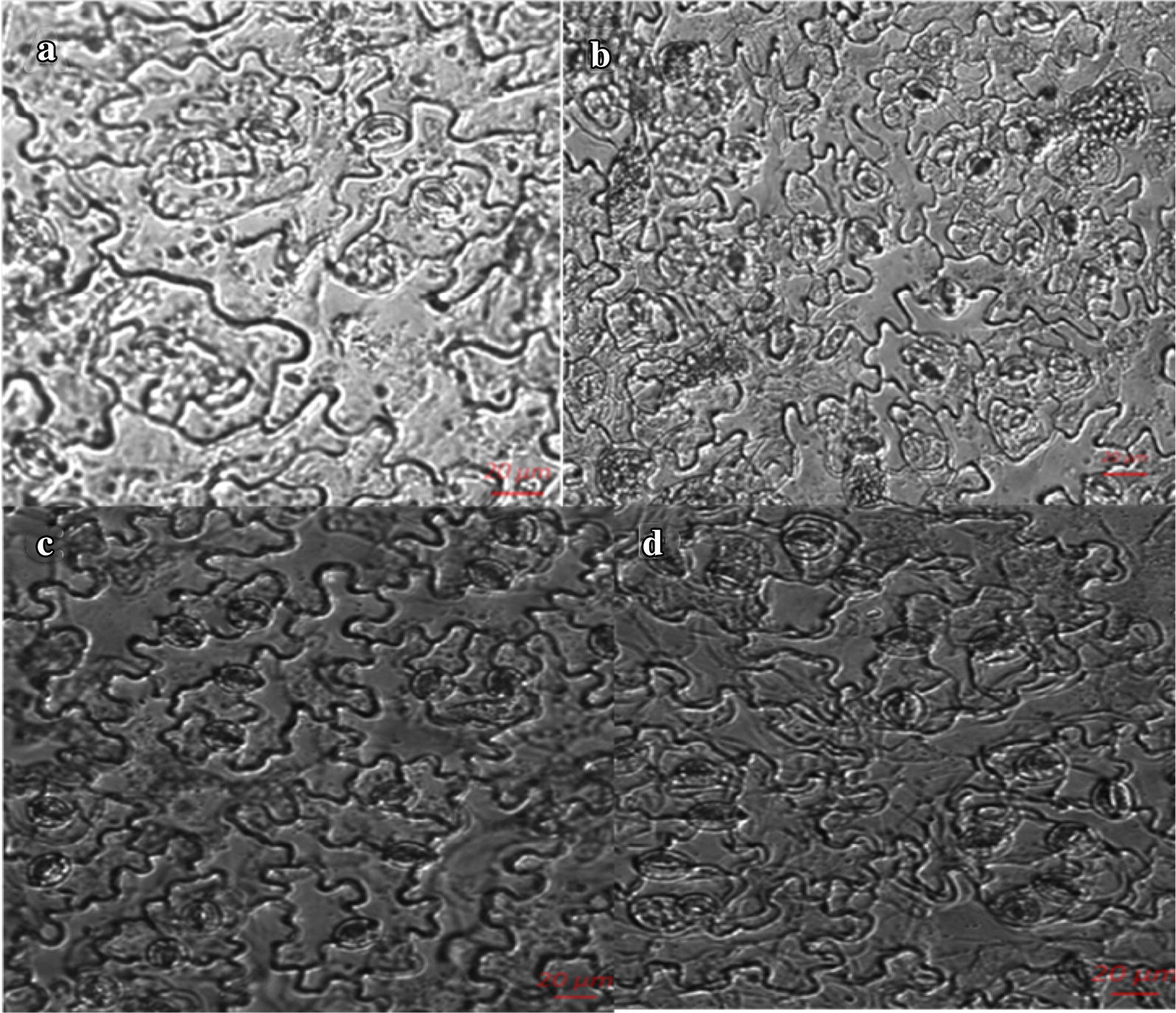
Epidermal cells in mature leaves (**a**) Self-grafting of *A. thaliana* (A/A), (**b**) Grafted seedlings of A/E, (**c**) Self-grafting of *E. salsugineum* (E/E), (**d**) E/A grafted seedlings

The size, shape, and leaf pigmentation pattern of salt cress and *Arabidopsis* were markedly affected by grafting. The results showed that the growth of the aerial part of the A/E plants was slightly depressed; the longer the vegetative growth stage was, the more obvious the difference was in the plant sizes. Grafting resulted in smaller *A. thaliana* parts in A/E grafts compared to the A/A plants (Fig. 3a). In contrast, larger *E. salsugineum* shoots (E/A) were observed when compared to the E/E plants (Fig. 3b). Furthermore, the leaf pigmentation pattern of the scion in A/E grafts was similar to that of the E/E homografts. Similarly the leaf color of the *E. salsugineum* part was similar to that of the A/A grafts. These interesting phenomena revealed that some traits associated with morphological characteristics, growth, and development characteristics were changed by grafting. Interestingly, we found that the rootstock has a profound effect on the growth and developmental characteristics of the scion. It has been suggested that grafting can be used to study the transduction of plant signals and interaction between above- and below-ground parts. Considering the high salt tolerance of *E. salsugineum*, it is worth studying the performance of A/E and E/A grafts under saline condition in the future. We observed no significant difference between the plants during the first 2 weeks after being transferred to a hydroponic system, but the difference in growth was more apparent over time. After 2 months of culture in the hydroponic system, *Arabidopsis* was significantly smaller than the corresponding self-grafted seedlings, when *A. thaliana* as scions were grafted onto *E. salsugineum* rootstocks. Furthermore, the size of all the rosette leaves was smaller than that of the self-grafted seedlings (Fig. 3a) and the size of epidermal cells in mature leaves also smaller (Fig. 4a). The results were opposite when compared E/A grafts to the E/E plants (Figs. 3b, 4b).

### Ion content determination

We employed ICP-OES (inductively coupled plasma atomic emission spectroscopy) to determine the levels of six elements, viz., Ca, Fe, K, Mg, Na, and Zn, in samples obtained 2 months after grafting. ICP-OES is one of the most precise multi-element analysis technique for trace and minor metals [32] and has the advantage of measuring both macronutrients and micronutrients with a wide range of concentrations in plants. The concentration of minerals in grafted samples (Table 3) is expressed in micrograms per gram. The ion contents in *A. thaliana* and *E. salsugineum* clearly changed to varying degrees after they were grafted together. Our original intention of the study was to determine how salt tolerance changes when a halophyte is grafted with a glycophyte. Therefore, we focused on changes in sodium and potassium ion concentrations. The ratio of potassium to sodium changed to some extent after grafting.

**Table 3:** The concentration of minerals in grafted samples

Grafts	Ion					
	Potassium	Sodium	Calcium	Magnesium	Iron	Zinc
A/A-shoot	12,858.32 ± 931.80	1204.88 ± 80.59	36,524.40 ± 874.03	7420.49 ± 435.10	141.77 ± 14.42	38.94 ± 3.21
A/E-shoot	10,106.16 ± 1205.97^d^**	1397.9 ± 247.91	39,942.75 ± 202.28	6138.81 ± 461.11	135.00 ± 20.06	35.38 ± 1.70
E/A-shoot	14,868.31 ± 695.20	707.89 ± 24.66	37,169.66 ± 1662.76	4276.28 ± 546.09	118.95 ± 34.96	25.63 ± 1.49
E/E-shoot	12,406.83 ± 749.57^a^*	893.8 ± 95.48	27,259.93 ± 1500.16^a^*	3777.52 ± 421.56	121.62 ± 20.87	41.69 ± 3.55^a^**
A/A-root	10,171.13 ± 528.54^b^*	1396.41 ± 60.72^b^*	2818.28 ± 168.40^b^*	833.00 ± 20.42^b^**	300.34 ± 12.82^b^*	12.42 ± 2.07^b^**
E/A-root	7569.79 ± 531.27	1709.06 ± 133.45	3120.19 ± 964.76	618.87 ± 61.50	375.73 ± 22.73	32.06 ± 3.22
A/E-root	7748.17 ± 409.96	1528.33 ± 160.85	3328.57 ± 497.91	1700.96 ± 116.09	197.54 ± 27.08	21.87 ± 3.73
E/E-root	4163.79 ± 210.98^c^**	861.3 ± 46.00^c^**	2617.92 ± 458.93^c^*	1523.01 ± 160.50	196.75 ± 10.20	43.02 ± 6.34^c^*

Results expressed as mean ± standard deviation (n = 3)

* P < 0.05; ** P < 0.01

^a^E/A-shoot compare with E/E-shoot; ^b^E/A-Root compare with A/A-Root; ^c^A/E-Root compare with E/E-Root; ^d^A/A-shoot compare with A/E-shoot

## Discussion

In addition to grafting methods and seedling age, smoothness of cutting surface, cutting site and length of illumination will affect the success of grafting.

### Cutting blade and supports

**Fig. 5:**
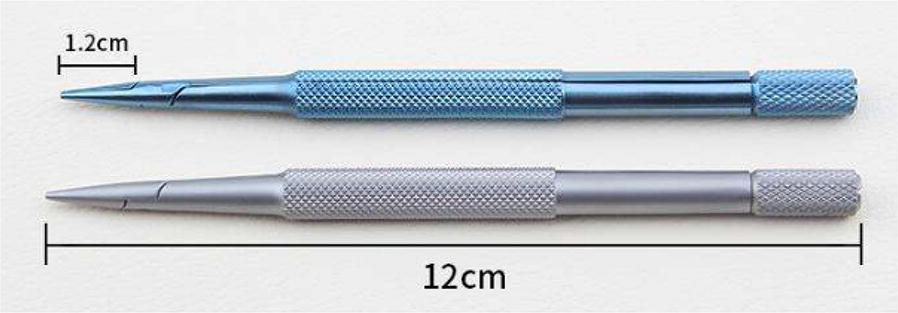
Blade holder

The smoothness of the cutting surface is one of the key factors that determine the success rate of grafting. Different blades including Swann medical surgical blades #11 and #22 (Swann-Morton), ordinary single edge razor blade (Shanghai Gillette), and stainless steel double-edged blade razor (Shanghai Gillette) were tested in this study. We found that double-edged blade razor worked well. Its knife edge is sharp and thin, and it is good for a smooth cutting surface, causing minimal damage to the meristem and thus reducing the formation of adventitious roots and improving the survival rate of grafted seedlings. With this approach, the injury to the cut surface is minimized, which help with the quick recovery of rootstock and scion after butt-jointing. This can be attributed to the fact that, as indicated by Turnbull [25], the rapid formation of graft union diminished adventitious rooting. The double-edged blade can be re-used more than 100 times. In order to make the blade last longer and reduce the trouble of replacement, we sterilized it by soaking it in 70% alcohol instead of flaming it. However, it is important to replace the blade when it loses sharpness. We fixed the blade to a blade holder (Fig. 5), which is more conducive for cutting and for reducing contamination.

Neither link support nor cutting support was employed in this study. The scion and stock were placed together on the medium without any supporting elements. The concentration of the agar, i.e., 1.0%, was sufficient to harden the medium to serve as the support for cutting the material. After cutting on the medium with 1.0% agar, no collar supports were used between scion and stock, allowing the healing happen in vertically oriented plates. This significantly simplifies the grafting process and shortens the time. With some practice, as many as over 30 heterografts or more than 50 homografts per hour can be achieved. This simplified method also reduces contamination rate often seen with complicated operation steps and ensure the success rate of grafting.

### Cutting position

It is noteworthy that the cutting position also affects the success rate of grafting. The survival rate of grafted plants was higher when the cutting site was in the upper half of the hypocotyl. We generally cut at one-fourth of length from the top of the *A. thaliana* hypocotyl as scion, and then grafted it onto the prepared *E. salsugineum* rootstock. Adventitious roots could appear above the graft junction and should be removed.

### Plate orientation and day light length

**Fig. 6:**
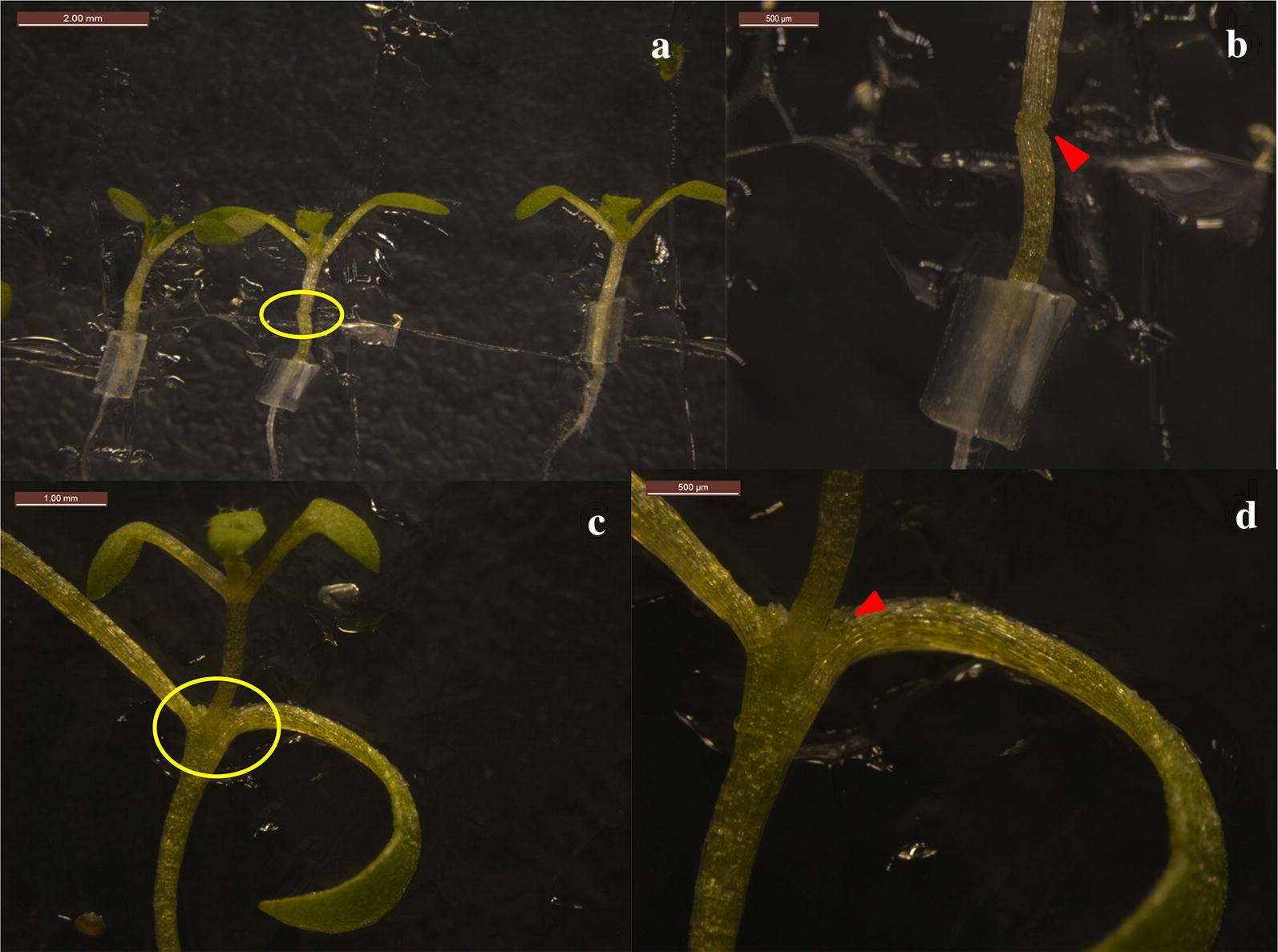
The grafting sites (**a**) succeed grafted seedlings of A/A. **b** Enlargement of yellow ellipse label in (**a**). **b** succeed grafted seedlings of A/E. **d** Enlargement of yellow ellipse label in **c**

Unlike other methods, we did not use support at the junction between the scion and the rootstock. They were placed together on the surface of the medium and allowed to heal in vertically oriented position. The surface of excised tissues should be in tight contact. It is easy to distinguish scions from stocks, because the grafting sites are clearly visible (Figs. 2, 6).

Quick operation is also crucial In order to keep the incision fresh. Typically 8–10 grafts were prepared on each petri dish, and then sealed with Parafilm to maintain high humidity but without contamination. It is necessary to handle the petri dishes with grafts gently so that scions from rootstocks were not dislodged.

We used SD light conditions because the hypocotyls of *A. thaliana* and *E. salsugineum* elongate faster compared to under LD light condition. Long hypocotyls are not only useful for cutting, but also convenient for grafting on a plate. Furthermore, SD light cycle is necessary for the growth of seedlings after grafting. It is well known that SD conditions promote the vegetative phase of *A. thaliana*, which help connect the calluses at the junction and avoid adventitious root growth.

After the grafting the plants were grown and allowed to recover from the grafting procedure under SD conditions in a Percival growth cabinet. After confirming that the grafting is successful under this condition (approximately 8–10 days after the grafting procedure), the grafts were transferred to a hydroponic system, and further experiments were also performed under SD light condition, in order to maintain the growth rate of grafted plants relatively consistent in later stages.

Seeds can be germinated under LD or SD light conditions, but after grafting the seedlings should be cultured under SD. In the cut-in grafting method, SD light condition can not only improve the survival rate of grafted plants, but also benefit the growth of grafted seedlings at later stages of development. This is because the growth cycle of *A. thaliana* is shorter compared to *E. salsugineum*. After the cut (i.e. injury), the flowering time of *A. thaliana* will be further promoted. Indeed LD light conditions lead to small plants and early flowering of the grafted seedlings, and early completion of the life cycle. SD light conditions prolong the vegetative phase and give the grafts sufficient time to recover from the injury.

## Conclusions

*Arabidopsis thaliana* is a popular model for plant molecular biology studies, but it is a glycophyte. Although many genes involved in plant stress signaling have been identified using this system, manipulating none of them is sufficient to bring salt tolerance of *A. thaliana* to the same level as *E. salsugineum*, which was proposed as a model halophyte ~ 20 years ago. The study of physiological and molecular mechanisms underlying salt tolerance in *E. salsugineum* will enable better understanding of the salt tolerance mechanism of plants. The reports on grafting across different genera is limited. This is the first report on inter-generic mutual grafting between salt cress and *Arabidopsis*. The grafting methods described here will guarantee plant grafting for the study of shoot–root interaction mechanisms, especially the molecular mechanism underlying long range salt transport in plants.

## Data Availability

The datasets used and/or analyzed during the current study are available from the corresponding author on reasonable request.
